# Postoperative Bleeding Risk for Oral Surgery under Continued Clopidogrel Antiplatelet Therapy

**DOI:** 10.1155/2015/823651

**Published:** 2015-01-06

**Authors:** Alexander Gröbe, Meike Fraederich, Ralf Smeets, Max Heiland, Lan Kluwe, Jürgen Zeuch, Martina Haase, Johannes Wikner, Henning Hanken, Jan Semmusch, Ahmed Al-Dam, Wolfgang Eichhorn

**Affiliations:** ^1^Department of Oral and Maxillofacial Surgery, University Medical Center Hamburg-Eppendorf, Martinistraße 52, 20246 Hamburg, Germany; ^2^Department of Oral and Maxillofacial Surgery, General Hospital Balingen, Tuebinger Straße 30, 72336 Balingen, Germany

## Abstract

*Object.* To determine the incidence of postoperative bleeding for oral osteotomy carried out under continued monoantiplatelet therapy with clopidogrel and dual therapy with clopidogrel/aspirin. *Design.* Retrospective single center observatory study of two study groups and a control group. *Methods.* A total of 64 and 60 oral osteotomy procedures carried out under continued monoclopidogrel therapy and dual clopidogrel/aspirin therapy, respectively, were followed for two weeks for postoperative bleeding. Another 281 similar procedures were also followed as a control group. All oral osteotomy procedures were carried out on an outpatient basis. *Results.* We observed postoperative bleeding in 2/281 (0.7%) cases in the control group, in 1/64 (1.6%) cases in the clopidogrel group, and in 2/60 (3.3%) cases in the dual clopidogrel/aspirin group. The corresponding 95% confidence intervals are 0–1.7%, 0–4.7%, and 0–7.8%, respectively, and the incidences did not differ significantly among the three groups (*P* > 0.09). Postoperative hemorrhage was treated successfully in all cases with local measures. No changes of antiplatelet medication, transfusion, nor hospitalisation were necessary. No major cardiovascular events were recorded. *Conclusions.* Our results indicate that minor oral surgery can be performed safely under continued monoantiplatelet medication with clopidogrel or dual antiplatelet medication with clopidogrel/aspirin.

## 1. Introduction

Clopidogrel is a common antiplatelet agent used as an alternative for aspirin or in dual antiplatelet therapy with aspirin [1–3]. Clopidogrel irreversibly inhibits adenosine diphosphate, which is necessary for platelet aggregation while aspirin works through inactivation of the enzyme cyclooxygenase. Both drugs prevent clot formation for the lifetime of the platelet, which is 9–11 days [4, 5].

When patients under such antiplatelet therapy need surgery, the surgeon is confronted with the choice of interrupting the therapy, which increases the risks of thrombosis, or continuing the medication, which on the other hand increases the risk of hemorrhage. Published studies commonly recommended continuation of antiplatelet drugs for minor oral surgery [3, 6, 7]. However, a perioperative interruption of antiplatelet medication is still frequently practiced for dental procedures [8]. In particular, clopidogrel, either for single or dual therapy, is feared for exposing patients to a high risk of bleeding [9] since it takes 5–10 days for full recovery of platelet activity after withdrawal.

The purpose of this study is to evaluate the postoperative bleeding rate for oral osteotomy and other invasive oral procedures under continued monoclopidogrel therapy or dual clopidogrel/aspirin therapy. We focus on oral osteotomy because this procedure involves invading the bone and therefore has a higher bleeding risk than other minor oral procedures such as tooth extraction.

## 2. Materials and Methods

A total of 405 oral osteotomy procedures were followed for 2 weeks. Some patients underwent multiple procedures. Each procedure was counted as an independent case (not the number of the patients) and subsequent analysis was based on this definition. A total of 64 procedures were carried out in patients who were under monoantiplatelet treatment with clopidogrel (75 mg/day) and another 60 procedures in patients under dual antiplatelet medication with clopidogrel (75 mg/day) and aspirin (100 mg/day). In all these 124 cases, medication was continued as usual and no change, neither interruption, reduction, nor bridging, was conducted. The rest of 281 osteotomy procedures were carried out in patients who were not under any anticoagulation or antiplatelet treatment.

All procedures were carried out by the senior author himself under local anaesthesia with articaine 4% and epinephrine 1 : 200.000 (Ultracaine D-S, Sanofi-Aventis) on an outpatient basis. In case of contraindications to epinephrine, scandicain 4% (Scandicain 4%, AstraZeneca) was used. After osteotomy, meticulous curettage of the extraction socket was performed and all of granulation tissue removed. Local hemostasis was achieved with a collagen fleece; wound closure was carried out with sutures and an acrylic splint. All patients received a single shot of 1000 mg amoxycillin as a prophylactic antibiotic measure. Postoperative pain was treated with ibuprofen 400 mg every 6 hours for 3 days or further if needed. Patients were routinely seen on the first, third, seventh, tenth, and 14th day. A bleeding was defined as an event that required additional surgical intervention.

Patient data were recorded using Evident (Evident, Bingen, Germany) and evaluated using SPSS. Confidence interval for bleeding incidence was calculated for 95% level [10].

## 3. Results

Postoperative bleeding was generally rare. Among the 281 osteotomy procedures for patients not under any antiplatelet medication, bleeding occurred only in 2 (0.7%) cases with a 95% confidence interval of 0.2–2.6%. For the 64 and 60 procedures under continued mono- and dual antiplatelet therapy, postoperative bleeding occurred in 1 (1.6%) and 2 (3.3%) cases, with 95% confidence intervals of 0.3–8.3% and 0.9–11.3%, respectively. The bleeding rates were not significantly higher in the mono- and dual antiplatelet treatment groups compared to that in the group of nonanticoagulation (Table 1). However, the statistical powers were only 0.18 and 0.45.

All bleedings occurred within 48 hours after the operation. For 4 out of the 5 bleeding cases, local measures with pressure on the osteotomy wound with methyloxycellulose was sufficient to achieve hemostasis. For the last case, fibrin glue (Tissucol Duo S, Baxter) was applied in addition to the methyloxycellulose. No change of the antiplatelet medication, no transfusion of blood, nor hospital admittance was necessary.

## 4. Discussion

The postoperative bleeding rate for osteotomy in the present study was generally low and was only slightly increased for cases under continued mono- and dual clopidogrel therapies (from 0.7% to 1.6% and 3.3%, resp.). However, due to the small sample size, especially that of the two therapy groups, the statistical powers were extremely low (0.18 and 0.45). The results of the present study are therefore of preliminary nature. Due to the small sample size, the data are not sufficient for detecting small differences in postoperative bleeding rates. At a 95% confidence level, the ranges of the bleeding rates can be estimated as below 8.3% and 11.3% for the mono- and dual therapy cases, respectively. With larger sample size in future studies, we expect to narrow down these ranges.

We have demonstrated that the bleedings were all manageable with simple local measures without stopping or modifying the anticoagulation therapy. This may ease the fear of lacking immediate countermeasures for clopidogrel to some extent [6, 11, 12].

For minor oral surgery such as teeth extraction, increasing data suggests that continuing antiplatelet medication during oral surgery does not increase the bleeding risk notably whereas the role of local hemostasis is emphasized [13, 14]. However, only very limited cases involved clopidogrel [15–17]. For example, Park et al. [18] observed 1.7% (1/59) excessive intraextraction bleeding under continued dual antiplatelet therapy but failed to follow up the patients for subsequent bleeding incidences. Girotra et al. [19] reported 5.2% immediate postoperative bleeding for oral surgery (mostly dental extraction) with continued monoclopidogrel and 7.9% for dual clopidogrel therapies. Oral osteotomy is more invasive than minor oral procedures such as standard teeth extraction. Nevertheless, the postoperative bleeding rates of 1.6% and 3.3% under continued mono- and dual clopidogrel therapy in our study are comparable or even below the already published ones.

We have recently followed bleeding for similar procedures under continued aspirin and phenprocoumon therapy and obtained the rates of 1.6% and 7.4%, respectively. Our findings confirm that the postoperative bleeding rates under continued anticoagulation therapy vary depending on the target of the therapy and the used reagents [20–22]. In case of continued phenprocoumon therapy, surgeons should be more cautious. By contrast, continuing antiplatelet therapy with aspirin and clopidogrel is likely safer.

In any case, preventive measures are recommendable for oral osteotomy under continued antiplatelet therapy. For example, we close the wound using a collagen sponge and sutures and further covered the site with an acrylic splint. Others pointed out possible timing effect and suggested the beginning of the week and morning hours for the surgery [13, 23]. Since most bleeding events are within 2 days after the surgery, a 24-hour hotline for two days may provide an effective measure ensuring quick response in case of bleeding.

## 5. Conclusion

Our results suggest that, despite its more invasive nature, oral osteotomy can be performed safely under continued monoantiplatelet medication with clopidogrel or dual antiplatelet medication with clopidogrel/aspirin.

## Figures and Tables

**Table 1:** Clinical features and bleeding rates in control and antiplatelet groups.

Features and Parameters	Antiplatelet medication			Chi-test, *t*-test (control versus mono-/dual therapy group)	
Group	Control	Clopidogrel	Clopidogrel/Aspirin	Significance *P*	Power
Number of cases	281	64	60		
Postoperative bleeding					
Case	2	1	2		
Rate (95% confidence)	0.7% (0.2–2.6%)	1.6% (0.3–8.3%)	3.3% (0.9–11.3%)	0.46/0.14	0.18/0.45
Age (years)	60.7 ± 6.3	67.9 ± 14.6	64.0 ± 10.8	<0.00/<0.00	0.98/0.74
Male/female	129/152	35/29	44/16	0.21/0.00	0.24/0.98
Numbers of teeth	1.7 ± 1.8	1.5 ± 0.8	1.5 ± 1.1	0.32/0.38	0.39/0.30
Dose		75 mg/day	75 mg + 100 mg/day		
Indication for antithrombotic medication					
Apoplexy + TIA		6	7	0.15	
Stent + bypass		16	28		
Myocardial infarction + other heart disorders		12	13		
Others		3	3		
Unknown		27	9		
